# Epidemiology, Prevention and Control of Foodborne Microbial Pathogens

**DOI:** 10.3390/microorganisms13071435

**Published:** 2025-06-20

**Authors:** Gabriel Augusto Marques Rossi, Juliano Gonçalves Pereira, Marcia Nitschke

**Affiliations:** 1 Department of Veterinary Medicine, University Vila Velha (UVV), Av. Comissário José Dantas de Melo, n.21, Vila Velha 29102-920, ES, Brazil; 2 Department of Animal Production and Preventive Veterinary Medicine, School of Veterinary Medicine and Animal Science, São Paulo State University (UNESP), Prof. Dr. Walter Mauricio Correa Street, Unnumbered, Botucatu City 18618-681, SP, Brazil; juliano.pereira@unesp.br; 3 São Carlos Institute of Chemistry (IQSC), University of São Paulo, Trabalhador São-Carlense Av., 400, P.O. Box 780, São Carlos 13566-590, SP, Brazil; nitschke@iqsc.usp.br

Foodborne diseases are caused by the consumption of food or water contaminated with bacteria and their toxins, viruses, parasites, or chemical substances. This concerning public health issue has an important socioeconomic impact, leading to pressure on healthcare services, reduced workforce productivity, and negative effects on sectors such as international trade. Each year, approximately one in ten individuals worldwide becomes ill due to the consumption of contaminated food, resulting in more than 420,000 deaths, mainly affecting children under the age of five years old (around 125,000 of these fatalities annually) [1]. In Brazil, where the Guest Editors of this Special Issue are based, a total of 6874 outbreaks caused by contaminated food or water were reported to the Ministry of Health between 2014 and 2023, resulting in 110,614 cases of illness, 12,346 hospitalizations, and 121 deaths [2]. Clearly, despite the relevance of these data, it is well known that they do not fully reflect the true incidence of these diseases. This is primarily because most cases go under-reported or undetected, as they often present with mild and self-limiting symptoms. As a result, affected individuals typically do not seek medical attention, which prevents the proper investigation and official reporting of the cases.

According to Scharff (2015), in 2013, the average cost per case of foodborne illness in the United States ranged from USD 1149 to USD 1925. The total economic burden was estimated to be between USD 54.9 billion and USD 92.0 billion, depending on the economic model applied for the analysis [3]. In this country, during 2019, it was estimated that nearly 9.9 million cases of foodborne illness originated from domestic sources and were attributed to certain primary pathogens: *Campylobacter* spp., *Clostridium perfringens*, invasive *Listeria monocytogenes*, norovirus, nontyphoidal *Salmonella* serotypes, and Shiga toxin-producing *Escherichia coli* (STEC). When *Toxoplasma gondii* was included in the analysis, the total burden rose to approximately 53,300 hospital admissions and 931 deaths. Among these, norovirus was the most frequently identified agent, followed by *Campylobacter* and nontyphoidal *Salmonella*. Despite being less common than norovirus, *Salmonella* was the leading cause of mortality linked to foodborne infections [4]. More recently, these values were updated, and in 2023, the cost of foodborne diseases in the United States was estimated to be approximately USD 75 billion. The etiological agents responsible for the greatest economic impact were nontyphoidal *Salmonella*, *Campylobacter*, *Toxoplasma*, and *L. monocytogenes* [5]. In Denmark, just seven pathogens—*Campylobacter* spp., *Salmonella* spp., Shiga toxin-producing *E. coli* (STEC), *Yersinia enterocolitica*, *L. monocytogenes*, Norovirus, and Hepatitis A virus—were responsible for 268,372 cases, 98 deaths, and 3121 disability-adjusted life years (DALYs) in a single year. These infections resulted in a total cost of 434 million euros in a country with a population of 5.8 million [6].

Thus, it becomes clear that controlling the occurrence of foodborne microbial pathogens throughout the entire food production chain is of utmost importance, as food products can become contaminated at any stage of production through a wide range of sources. This includes implementing targeted strategies at the primary production level on farms, ensuring proper handling and safety measures during industrial processing, maintaining hygiene and safety standards at the point of sale, and promoting safe food preparation practices in households. This Special Issue, titled “Epidemiology, Prevention and Control of Foodborne Microbial Pathogens” was developed with the aim of compiling scientific articles that provide valuable insights into the epidemiology of major foodborne pathogens. Additionally, it highlights preventive measures targeting various stages of the food production chain, with the goal of reducing the incidence and public health impact of the diseases caused by these bacteria. A total of seven contributions were accepted for publication after undergoing the peer review process. These articles, which are briefly summarized below, address key aspects related to *L. monocytogenes*, *C. perfringens*, *Salmonella* spp., *E. coli*, and *Campylobacter* spp.

Two contributions focused on *L. monocytogenes*, an important pathogen capable of causing gastroenteritis or severe clinical conditions such as meningitis and intrauterine infections. The first contribution, published by Papadochristopoulos et al., explored the potential of natural antimicrobials to control *L. monocytogenes* in vacuum-packed beef burgers. The antimicrobials tested included carvacrol; essential oils from thyme, rosemary, clove, and cinnamon; hop extract; cranberry extract and pomace; propolis extract; and chitosan derived from both shrimp and mushrooms. The authors also assessed the sensory impact of these treatments on the product. The findings highlighted chitosan’s promising effectiveness in controlling *L. monocytogenes* in beef burgers, with particular emphasis on the advantages of using mushroom-derived chitosan [7]. The second manuscript, authored by Vasileiadi et al., assessed the growth potential of *L. monocytogenes* in raw sea bass (*Dicentrarchus labrax*) fillets using a challenge test. Based on their results, the authors suggested that raw fish products used in the preparation of ready-to-eat foods, such as sushi and various seafood dishes, should be included in the Safety Criteria Category of Regulation (EC) No. 2073/2005 as “ready-to-eat food able to support the growth of *Listeria monocytogenes*” [8].

Two noteworthy studies focused on *C. perfringens*, a spore-forming bacterium associated with foodborne illness. In the first study, Alfattani et al. tested 15 natural compounds for their ability to inhibit spore development. Among these, garlic and onion juice, as well as undiluted essential oil components from clove, rosemary, and peppermint, demonstrated the highest effectiveness. These selected substances were subsequently examined during different phases of the bacterium’s life cycle, including spore germination, outgrowth, and vegetative cell development, using both microbiological media and chicken meat as test systems. While some of the natural agents successfully inhibited bacterial growth in vitro, their application to chicken meat contaminated with *C. perfringens* spores did not yield comparable antimicrobial effects [9]. Bansal et al. also investigated *C. perfringens*, providing valuable insights into its pathogenic mechanisms. Their study demonstrated that the mechanistic target of the rapamycin (mTOR) signaling pathway plays a key role in mediating *C. perfringens*-induced ileitis. Moreover, the authors showed that a combined therapeutic approach using mTOR inhibitors and deoxycholic acid (DCA) enhanced the effectiveness of treatment, mitigating both necrotic enteritis-associated intestinal inflammation and the resulting loss of body weight [10].

Finally, the Special Issue also addressed other important foodborne pathogens, including *Salmonella* spp., *E. coli*, and *Campylobacter*. Brito et al. investigated the presence of these microorganisms in broiler carcasses with or without visible fecal contamination during processing at a slaughterhouse. Their findings revealed that the mere absence of gastrointestinal content on carcasses does not guarantee microbiological safety. Even carcasses without visible contamination were found to carry high bacterial loads, including *Salmonella* spp. and *E. coli* [11].

The occurrence of *E. coli* was also explored in another study by Ribeiro et al., which focused on the raw milk fresh cheese production chain in Brazil. This research aimed to assess both the virulence and antimicrobial resistance profiles of the isolates. Pathogenic strains, including enteropathogenic (EPEC), Shiga toxin-producing (STEC), and extraintestinal pathogenic *E. coli* (ExPEC), were detected in both milk and cheese samples. A high prevalence of resistance was observed against several antibiotics, including nalidixic acid, ampicillin, kanamycin, streptomycin, sulfisoxazole, and tetracycline. In addition, various sequence types (STs) were identified, with serogroup O18 being the most frequently detected. Genetic and epidemiological analyses indicated the dissemination of these resistant and potentially pathogenic strains across different points of the production chain [12].

Lastly, a literature review authored by Veronese & Dodi focused on the microaerophilic bacteria *Campylobacter jejuni* and *Campylobacter coli*. The authors provided an in-depth overview of multiple aspects of these pathogens, including their morphological and taxonomic characteristics, epidemiology, pathogenic mechanisms, and virulence factors, as well as the clinical manifestations in human populations. The review also covered current approaches to diagnosis and treatment, along with the growing concern regarding antimicrobial resistance in these species [13].

As Guest Editors, we believe that the contributions compiled in this Special Issue offer valuable insights into a range of foodborne microbial pathogens, addressing not only their epidemiology but also prevention and control strategies. Reducing the presence of these microorganisms in food remains a major challenge, as contamination can occur at multiple stages along the production chains of various food types. Nevertheless, this effort is essential to ensure the delivery of safe food to the population while also minimizing losses for food industries and reducing public health costs.
